# Study protocol: non-displaced distal radial fractures in adult patients: three weeks vs. five weeks of cast immobilization: a randomized trial

**DOI:** 10.1186/1471-2474-15-24

**Published:** 2014-01-20

**Authors:** Abdelali Bentohami, Niels de Korte, Nico Sosef, Johan Carel Goslings, Taco Bijlsma, Niels Schep

**Affiliations:** 1 Department of Surgery, Spaarne Hospital, Hoofddorp, The Netherlands; 2 Trauma Unit Department of Surgery, Academic Medical Center, Amsterdam, The Netherlands

## Abstract

**Background:**

Up to 30% of patients suffer from long-term functional restrictions following conservative treatment of distal radius fractures. Whether duration of cast immobilisation influences functional outcome remains unclear.

**Methods/Design:**

The aim of the study is to evaluate whether the duration of immobilization of non or minimally displaced distal radial fractures can be safely reduced. We will compare three weeks of plaster cast immobilization with five weeks of plaster cast immobilization in adult patient with non or minimally displaced distal radial fractures.

Study design: a prospective randomized clinical trial.

Study population: adult (>18 years) (independent in activities of daily living) patients with a non/minimal displaced distal radius fracture (dorsal angulation <15°, volar tilt <20°, radial inclination >15°, ulnar positive variance <5 mm and an articular step off <2 mm).

Intervention: three weeks of plaster cast immobilization versus five weeks of plaster cast immobilization.

Main study parameters: primary outcome parameters: Patient related wrist evaluation (PRWE) Quick Disability of Arm, Shoulder and Hand (QUICKDASH) score after a one year follow-up, and secondary parameters: range of motion, pain level (VAS) and complications.

**Discussion:**

The expectation of this study is that shorter duration of plaster cast immobilisation is beneficial for the patient with a distal radius fracture. This risk of specific complications is low and generally similar in both treatment options. Moreover, the burden of the study is not much higher compared to standard treatment. Follow-up is standardized according to current trauma guidelines. Literature indicates that both treatment options from the study are accepted for displaced distal radius fractures. No clear advantage for one treatment options is found at present in the literature, although there is no level I evidence present. This trial will provide level-1 evidence for the comparison of consolidation and functional outcome between two treatment options for non-displaced distal radial fractures. The gathered data may support the development of a clinical guideline for conservative treatment of distal radial fractures.

**Trial registration:**

Netherlands National Trial Register NTR3552.

## Background

Fractures of the distal radius are common injuries and account for up to 15% of all extremity fractures [1]. Most of these patients can be treated non-operatively in a plaster of Paris, with excellent functional results [2,3]. Usually a immobilization period of four till six weeks is preferred. The duration of immobilisation has been questioned earlier in literature. Some authors believe that three weeks is long enough [4-6], while others consider one week of immobilization is sufficient [7]. Other authors even consider that there is no need for plaster immobilsation in case of a non-displaced distal radial fracture [8,9].

The duration of immobilization of distal radius fractures depends on whether these fractures can displace into an unacceptable position. Most radial fractures are liable to displace within the first two weeks [10], only 7% to 8% displace after this time [10,11], and none after six weeks [12]. A minimum period of three weeks of immobilization seems safe.

Two randomized controlled trials compared three-weeks with five-weeks immobilization in a plaster back slab, in a total of 133 patients [5,6]. Most patients had minimally displaced distal radial fractures. Both trials found no significant differences in either anatomical or functional outcomes after 9 months [5] and one year [6] follow up. Separate data for complications were not given. Jensen’s RCT in 1997 showed in 62 patients with an undisplaced extra-articular distal radius fracture that even one week of immobilization did not differ from three weeks of immobilization when comparing radiographic results or functional outcome defined by the, Gartland and Werley score after 26 weeks [7]. Separate results for complications were not provided.

The studies mentioned above have their limitations in follow-up, modest patient groups or methods. In a review of Handoll 2008 concerning conservative interventions for treating distal radial fractures in adults it was also concluded that there is no scientific support for a preferred treatment strategy, including length of immobilization, for non-displaced distal radial fractures [13].

For example the Gartland and Werley score [14] was used to assess functional outcome in two studies [5,7]. This is the most commonly described instrument in the literature for evaluating outcome after wrist surgery, but it has not been validated so to date. In the other study radiography, wrist motion, grip strength and pain were measured after one year and no outcome measure instruments were used [6]. Thereby in all the studies serious complications were not reported separately, patient satisfaction and resource implications were rarely mentioned and there was an inadequate description of inclusion criteria. The variety of fracture classification systems, with associated issues of reliability and validity further complicates comparison between studies and their outcomes [15].

Obviously, the ultimate treatment is short, safe and leads to an early return of function. In achieving this, reduction of the immobilization period may be beneficial. The short period of immobilization could speed up the functional recovery or reduce the number of days absent from work. Since there is little knowledge about the best immobilisation period for non-displaced distal radial fractures, there is a need for a RCT. In this study functional outcome will be the primary outcome and this will be assessed using validated instruments, namely the Patient Rated Wrist Evaluation (PRWE) and the Disability of Arm and Shoulder (DASH) forms [16].

### Rationale for the trial

Nowadays, usually an immobilization period of four till six weeks is preferred. Despite the minimal evidence in literature this immobilization period can be questioned. A randomized clinical trial with sufficient power is needed to provide scientific support for a preferred treatment strategy for non-displaced distal radial fractures. The aim of this trial is to compare the results of three weeks of cast immobilization with five weeks of cast immobilization of non-displaced distal radial fractures with respect to functional outcome, the incidence of non-union, pain scores, and complications.

## Methods/Design

### Study design

**Figure 1:**
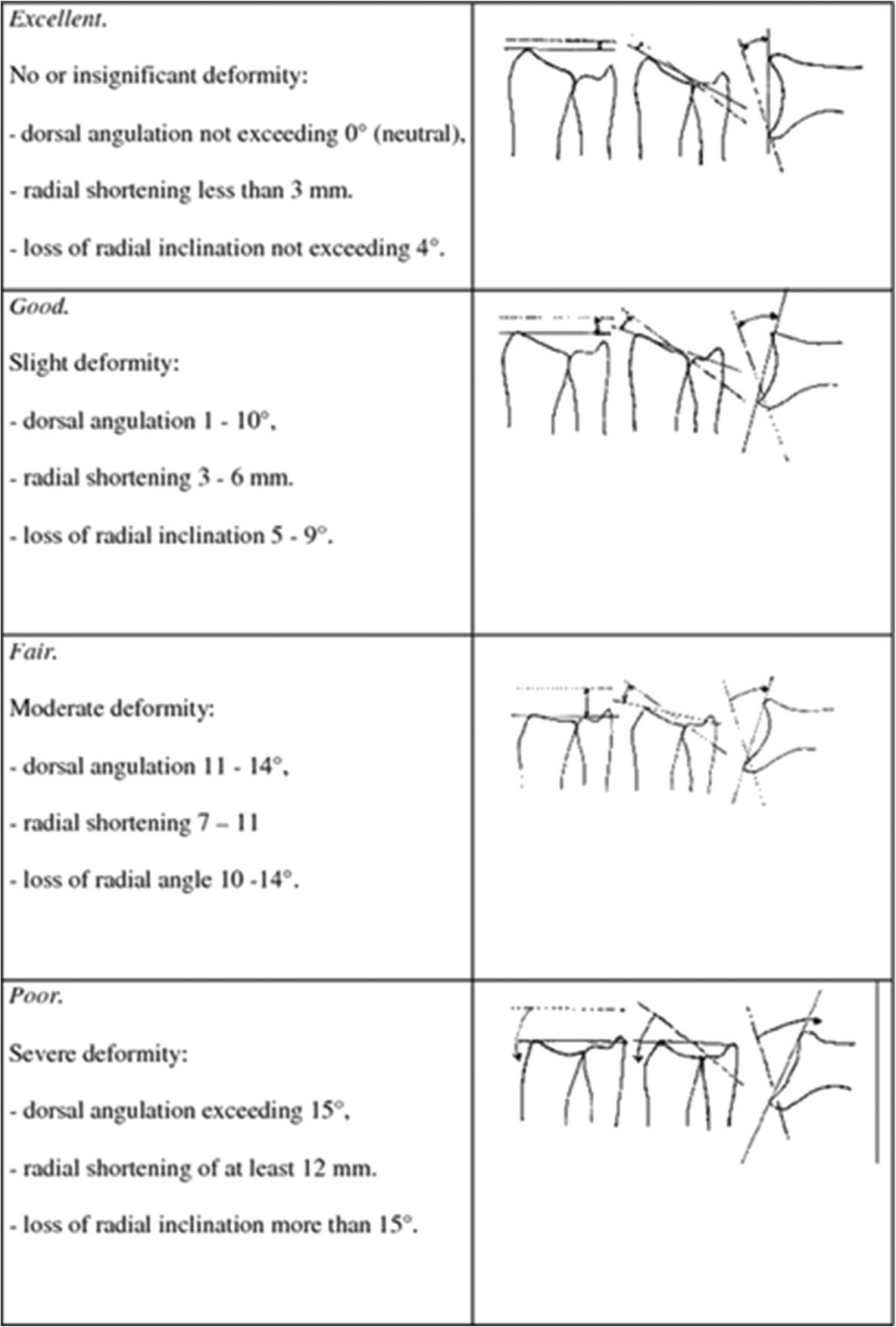
Randomization and inclusion period.

**Figure 2:**
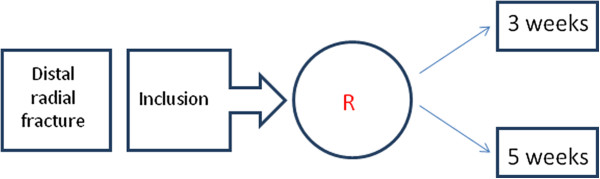
Follow-up.

This study will be conducted as a prospective randomized clinical trial (see Figures 1 and 2) in which three weeks of plaster cast immobilization is compared with five weeks of plaster cast immobilization. Patients will be treated in a short arm plaster cast. Patients will only receive physiotherapy if necessary. The study started, September 1^st^, 2012.

### Recruitment and consent

Patients with distal radius fractures will be initially managed on the emergency department. Using the criteria for displacement: distal radial fractures with initial dorsal angulation up to 15° and axial radial shortening of not more than 5 mm. Patients will be included if closed reduction of the distal radius fracture is not necessary according to these criteria for misalignment.

They will receive written information and a consent form from the attending physician, the clinical investigator or a research assistant. After providing informed consent, eligible patients will be randomized within one week. An independent research assistant will perform concealed permuted block randomization using a computer-generated randomization schedule after stratification for fracture type, gender and age. Allocation will be at random to four blocks.

### Blinding

#### *Functional status*

An independent research assistant will perform a blinded evaluation of the trial patients’ functional status.

#### *Radiographic outcome*

**Figure 3:**
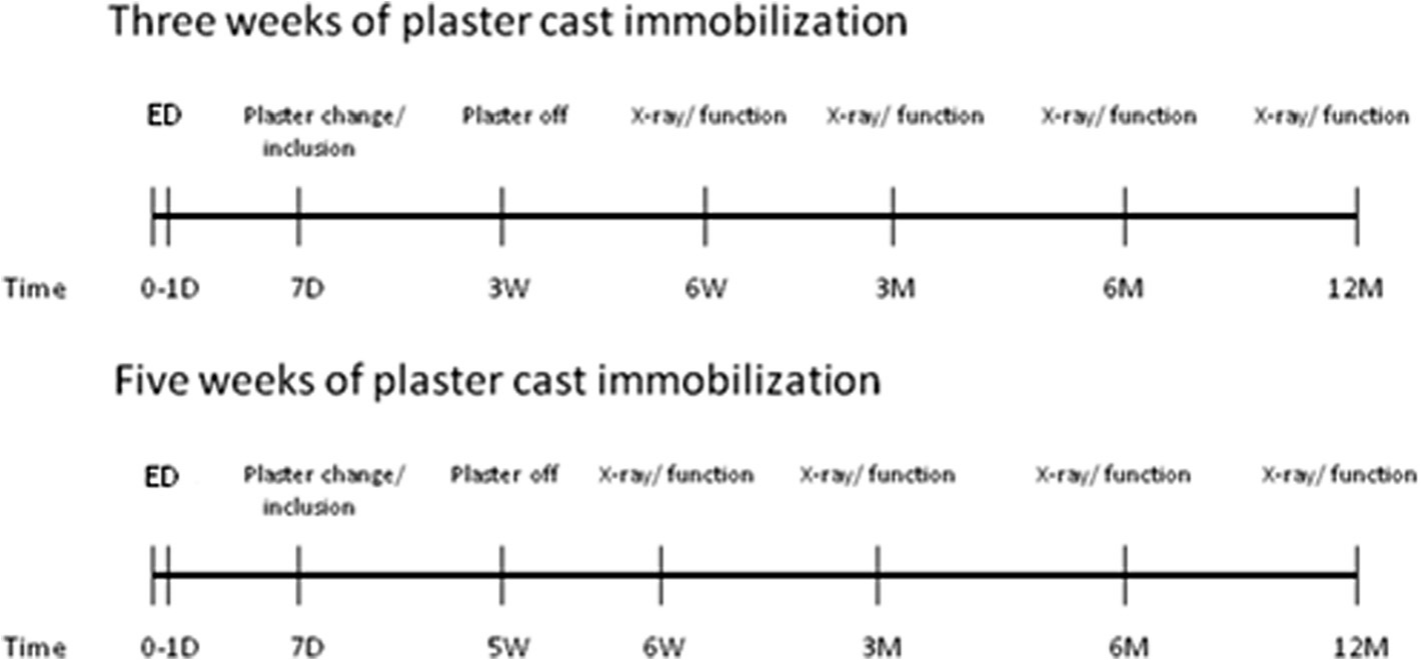
Anatomical radiological classification for distal radial fractures according to Lidström.

Radiographic evaluation of the alignment of the distal radius will be performed blinded for the intervention group. In addition to the treating physician, two independent experts will assess the Lidström score for the repeated X-rays of the wrist blinded from the first assessment (Figure 3).

### Bias prevention

To prevent bias stratification by age (younger and older than 60 years) and gender will be performed (Table 1).

**Table 1:** Stratification by gender and age (younger and older than 60 years)

	**Patient characteristics**		**Randomization**
List 1	Male	Median age -	ABAB AABB ABBA BABA BAAB
List 2	Male	Median age +	BAAB BBAA ABAB AABB ABBA
List 3	Female	Median age -	AABB ABBA BAAB BBAA BABA
List 4	Female	Median age +	ABBA BABA ABAB AABB AABB

A = 3 weeks, B = 5 weeks.

### Study population

The study population is defined as all adult patients with a non-displaced distal radius fracture. All patients should be independent in activities of daily living. Evaluation of eligible patients will take place either at the emergency department or at the outpatient department of the Spaarne Hospital, Hoofddorp. Patients are eligible using the following in- and exclusion criteria:

### Inclusion criteria

1. Age > 18 years.

2. Unilateral fracture of distal radius without misalignment (dorsal angulation <15°, Axial radial shortening <5 mm.

3. Independent for activities of daily living.

### Exclusion criteria

1. Fracture of contralateral arm.

2. Other fractures at the ipsilateral arm (excluded carpal fractures).

3. Pre-existent abnormalities fractured distal radius.

4. Open fractures.

5. Fracture that needs reduction.

### Outcome measures

The primary outcome measure is the Quick-DASH (Disabilities of the Arm, Shoulder and Hand) score [17], which reflects both function and pain and PRWE (Patient Related Wrist Evaluation) [18].

The DASH Outcome Measure is a validated 30-item, self-reported questionnaire designed to help describe the disability experienced by people with upper-limb disorders and also to monitor changes in symptoms and function over time [17].

The *Quick*-DASH is a shortened version of the DASH Outcome Measure. Instead of 30 items, the Quick-DASH uses 11 items (scored 1–5) to measure physical function and symptoms in people with any or multiple musculoskeletal disorders of the upper limb. The right and left elbow will be assessed separately. At least 10 of the 11 items must be completed for a score to be calculated. The scores will be transformed to a 0–100 scale for easy comparison. A higher score indicates greater disability.

Like the DASH, the *Quick*-DASH contains 2 optional modules to measure symptoms and function in athletes, performing artists and other workers whose jobs require a high degree of physical performance. These optional models are scored separately; each contains four items, scored 1–5. All items must be completed for a score to be calculated.

PRWE score is the most responsive instrument for evaluating the outcome in patients with distal radius fractures [18].

The secondary outcome measures are:

1. Range of motion.

2. Pain (assessed by the VAS scale).

3. Complications: dislocation, complex regional pain syndrome and mal/nonunion.

Pain level will be determined using a 10-point Visual Analog Scale (VAS), in which zero implies no pain and ten implies the worst possible pain.

ROM will be measured on both sides using a goniometer.

In addition to the outcome variables mentioned above, the following data will be collected:

a) Intrinsic variables (baseline data): age, gender, American Society of Anesthesiologists’ ASA classification, tobacco consumption, alcohol consumption, comorbidity, social status/household composition, dominant side, and medication use.

b) Injury related variables: affected side, mechanism of injury.

c) Intervention-related variables: time between injury and start of physical therapy and number of physical therapy sessions.

### Study procedures

After inclusion, all patients will be followed for one year in total. Clinical assessments will occur at the time of admission (ED), one week (3-10-day window), three weeks (11-28-day window) or five weeks (4-8-week window), six weeks (4-8-week window), three months (11-15-week window), six months (5-7-month window), and 12 months (12-14-month window) after start of treatment.

At each FU visit, the research coordinator or research assistant will ascertain patient status (i.e., secondary interventions, adverse events/complications, deaths) and will verify information within medical records.

At each FU visit, the patients will be asked to indicate the pain level on a VAS.

At each visit from six weeks onwards, the ROM of the wrist will be measured using a goniometer by a doctor blinded for the treatment method. In addition, patients will be asked to complete the questionnaires relating to disability (Quick-DASH score including optional modules, PRWE Score), and healthcare consumption.

Plain X-rays of the wrist will be made at the time of presentation in the hospital (ED), and at the follow-up visit after 6 weeks three months, six months and one year. The X-ray at 12 months will be taken in order to determine the grade of degenerative joint changes.

Time to define the presence of a malunion will be at three months. Intention-to-treat principle will be maintained (see Figure 2).

X-ray: control X-ray according to standard guidelines, assessment using Lidström score [19].

Function: functional assessment using functional outcome scores, range of motion, pain assessment using VAS scale.

ED = Emergency Department, D = days, W = weeks, M = months.

### Sample size

The primary outcome will be the QuickDASH score of which the minimal clinically important difference is 14 points. Based on a difference of 14 points, the sample size of 30 patients per treatment group was calculated with a power (1-β) of 80 percent and a type I error (α) of 5 percent, allowing for 10 percent drop-out. In total 70 patients will be included.

### Withdrawal of individual subjects

Subjects can leave the study at any time for any reason if they wish to do so without any consequences. The investigator can decide to withdraw a subject from the study for urgent medical reasons.

### Statistical analysis

#### *Descriptive statistics*

Data from the demographic data collection and the outcome parameters will be cleaned blindly from the treatment data. Data are presented as mean scores with 95% confidence intervals.

#### *Univariate analysis*

The analysis of this study will be carried out according to the intention-to-treat principle, i.e. the patients will remain in the group they will be randomly allocated to at baseline. Analysis of functional outcome will be assessed using repeated-measures analysis of variance (GLM 4) with the time as the within-group factor and the treatment as the between-group factor. Post-hoc analysis will be performed on the time of randomization. Group comparisons at the different time points will be made only when the overall repeated-measures tests are statistically significant. All scores will be tested for normality using the Kolmogorov-Smirnov test. Parametric variables will be compared using the Student’s t-test, while non-parametric and ordinal variables will be compared using the Mann–Whitney U statistic. Nominal variables will be compared across independent groups using the chi-squared test or Fisher’s exact test. Homogeneity of variance will be assessed using Levene’s test. Also a multiple regression will be performed. SPSS statistical software (version 11.0.1) will be used for the analysis, in which two-tailed P value < 0.05 will be considered significant.

### Ethical considerations

#### *Regulation statement*

The study was approved by the Regional Ethical Committee and will be carried out in compliance with the Declaration of Helsinki on ethical principles for medical research involving human subjects [20]. The Medical Ethics Committee Noord-Holland acts as central ethics committee for this trial (reference number M011-059; NL38449.09.11).

### Recruitment and consent

Patients with a distal radial fracture will be treated by the physician on call in the Emergency Department. The only difference is that duration of immobilization will be decided after randomization. Randomization will occur after informed consent.

### Administrative aspects and publication

#### *Handling and storage of data and documents*

The data will be coded by patient number. Research data will be stored in a database (PASW statistics 18 and Microsoft Excel), and will be handled confidentially and anonymously. Research data that can be traced to individual persons can only be viewed by authorized personnel. These persons are the members of the research team, members of the health care inspection, and members of the Medical Ethics Committee of the Academic Medical Center Amsterdam. Review of the data may be necessary to ensure the reliability and quality of the research. The handling of personal data is in compliance with the Dutch Data Protection Act (in Dutch: ‘Wet Bescherming Persoonsgegevens’, WBP) and the privacy regulation of the Academic Medical Center Amsterdam.

## Discussion

The duration of cast-immobilization for distal radial fractures remains a topic of debate. Currently, the decision for the duration of immobilization of distal radial fractures is predominantly based upon the personal preferences of the treating physician. The studies done for assessing the immobilization periods of distal radial fractures have their limitations of using a non-validated outcome score list, which makes it impossible to conclude with certainty shorter immobilization periods of distal radial fractures are preferred. Considering this statements, a new randomized trial with sufficient power is needed to provide evidence for a definitive, generally acceptable guideline for the treatment of non-displaced distal radial fractures. The results of this study will help to clarify the question if shorter periods of immobilizations are favorable in adult patients with conservatively treated distal radial fractures, thereby considering functional outcome, pain scores, incidence of non-union, and complications.

## Competing interests

The authors declare that they have no competing interests. No external funding was received for this study.

## Authors’ contributions

All authors participated in the design and the drafting of the manuscript. All authors have read and approved the final manuscript.

## Pre-publication history

The pre-publication history for this paper can be accessed here:

http://www.biomedcentral.com/1471-2474/15/24/prepub
